# Comprehensive analysis of immune-related gene signature based on ssGSEA algorithms in the prognosis and immune landscape of hepatocellular carcinoma

**DOI:** 10.3389/fgene.2022.1064432

**Published:** 2022-12-09

**Authors:** Liangliang Wang, Li Wang, Peihong He

**Affiliations:** ^1^ Chemoradiotherapy Center of Oncology, The Affiliated People’s Hospital of Ningbo University, Ningbo, China; ^2^ Department of Chemoradiotherapy, The Affiliated People’s Hospital of Ningbo University, Ningbo, China; ^3^ Department of General Surgery, Ningbo Yinzhou No. 2 Hospital, Ningbo, China; ^4^ Beilun Traditional Chinese Medicine Hospital, Ningbo, China

**Keywords:** immune-related gene, hepatocellular carcinoma, single sample gene set enrichment analysis, prognosis, immune infiltration

## Abstract

**Background:** Hepatocellular carcinoma (HCC) is a malignancy with a poor prognosis. This study aimed to distinguish patients with HCC having distinct tumour immune microenvironment (TIME) features and construct an immune-related gene signature (IRGs) to assess prognosis and provide a basis for personalised therapies.

**Methods:** Transcriptomic data of patients with HCC were downloaded from The Cancer Genome Atlas (TCGA) and Gene Expression Omnibus (GEO) databases. We assessed the immune cell infiltration in each HCC specimen using single sample gene set enrichment analysis (ssGSEA) and classified all patients with HCC into high- and low-immune clusters using a hierarchical clustering algorithm. The ESTIMATE and CIBERSORT computational methods were employed to verify the stability and effectiveness of the immune clusters. Subsequently, the differentially expressed genes (DEGs) of the high- and low-immune clusters and the immune-related genes intersected to obtain the immune-related DEGs. The least absolute shrinkage and selection operator (LASSO) was then employed to screen the optimal genes for the construction of a prognostic predictive signature and to divide patients into high- and low-risk subgroups. The predictive efficacy of the IRGs was further confirmed using Kaplan–Meier survival curves, univariate and multifactorial Cox regression and time-dependent ROC curves in the TCGA and GSE14520 validation cohorts. Furthermore, we developed a nomogram to predict the prognosis. Tumour mutation burden (TMB) was also analysed in the risk groups. Additionally, gene ontology and gene set variation analysis were used for biological function and pathway exploration. Lastly, drug sensitivity analyses were employed to investigate prospective therapeutics in the two risk populations.

**Results:** Immune cluster analysis based on ssGSEA could well distinguish the TIME characteristics of patients with HCC. The stromal score, immune score and ESTIMATE score were all lower in the low-immune cluster. Meanwhile, most of the immune checkpoint-related genes and HLA family genes were overexpressed in the high-immune cluster, suggesting that this cluster could be a beneficial population for immune checkpoint inhibitors therapy. There were 1,617 DEGs between the two immune clusters, of which 414 genes intersected with immune-associated genes. Furthermore, Cox regression analysis revealed 49 DEGs that were associated with survival. Then, 19 DEGs were screened using the LASSO algorithm for IRGs construction and patients were classified into high- and low-risk groups. Both the constructed signature and nomogram had good prognostic predictive efficacy. The signature-based risk score was an independent prognostic predictor in both the TCGA and GSE14520 cohorts. Additionally, there was no significant difference in TMB between the two risk populations. Lastly, the half-maximal inhibitory concentrations of certain chemotherapeutic and targeted therapeutic agents differed between the two risk groups.

**Conclusion:** Our study provides a personalized tool for predicting the prognosis and TIME landscape of HCC and a basis for developing personalised treatment regimens.

## Introduction

According to the GLOBOCAN 2020 report (Sung et al., 2021), hepatocellular carcinoma (HCC) accounts for 90% of all primary liver cancer and is one of the most common cancers in humans, characterized by insidious onset, poor prognosis and high mortality (Huang et al., 2020; Llovet et al., 2021). Despite advancements in diagnostic and therapeutic approaches for HCC, the prognosis for individuals with HCC remains poor, with an estimated 5-year survival rate of only 18% (Craig et al., 2020). Current systemic treatments, such as tyrosine kinase inhibitors (TKIs) and immune checkpoint inhibitors (ICIs), provide patients with more therapeutic choices; however, the high heterogeneity of HCC limits their efficacy and compromises the precision of prognostic prediction (Liu et al., 2016). Hence, sensitive and reliable indicators are needed to assess individualized differences and prognosis, thereby providing a reliable basis for the development of personalised patient treatment strategies.

With the rapid development of tumour immunology, ICI-based immunological therapies have achieved significant progress in the management of various tumours, providing novel options for the treatment of HCC (Jácome et al., 2021; Mamdani et al., 2022; Teixeira Farinha et al., 2022). The main advantage of immunotherapy is its relatively long-lasting effect; however, ICIs are only 12–20% effective compared to monotherapy (Abd El Aziz et al., 2020). In the CheckMate 459 (Yau et al., 2022) and KEYNOTE-240 (Finn et al., 2020b) clinical trials, ICSs monotherapy failed to meet the pre-defined clinical trial endpoints. In terms of combination therapy, the phase II study of nivolumab plus ipilimumab achieved results. The Investigator-assessed objective response rate (ORR) was 32% (Yau et al., 2020). In the phase III clinical trial (IMbrave150), the median OS was 19.2 months with atezolizumab plus bevacizumab and 13.4 months with sorafenib (Cheng et al., 2022). In addition, the combination of Pembrolizumab with Lenvatinib also showed efficacy in phase I clinical trials. The ORR was 36% and the median OS was 22 months (Finn et al., 2020a). Together, the above studies suggest the potential of immunotherapy in HCC.

The evolution of tumours is closely linked to the tumour immune microenvironment (TIME), which contains a variety of immune cells, stromal cells and cytokines, all of which can interact with tumour cells to form a highly complex system. Increasingly, studies have demonstrated that the regulation of immune system networks in TIME and tumour interactions have a significant impact on tumour prognosis and response to immunotherapy (Pitt et al., 2016; Binnewies et al., 2018; Galon and Bruni, 2019). In addition, TIME is a heterogeneous environment, and the TIME characteristics of individuals are often the result of randomisation across factors. Therefore, the development of novel and effective immune-related predictive biomarkers to analyse the correlation between TIME and HCC can help to determine the prognosis and TIME features of patients with HCC, allowing for the personalised selection of therapeutic strategies for ICIs.

The present study uses single sample gene set enrichment analysis (ssGSEA) and cluster analysis to classify patients with HCC into high- and low-immune clusters, whereas ESTIMATE and CIBERSORT analyses verify the stability and validity of the immune clusters. Furthermore, LASSO regression analysis establishes an immune-related gene signature (IRGs) to further validate the prognostic value of the IRGs in The Cancer Genome Atlas (TCGA) and GSE14520 independent cohorts. Additionally, we construct a nomogram to predict the prognosis of patients with HCC. Furthermore, Tumour mutation burden (TMB) also analyses the high- and low-risk groups. Gene set variation analysis (GSVA) and Gene Ontology (GO) analysis were also used for biological function and pathway exploration in this study. Finally, the IC50 of certain chemotherapeutic and targeted therapeutic agents were also analysed in high- and low-risk populations. Our results will not only help to determine the prognosis of clinical patient with HCC, but also provide a basis for the selection of personalized clinical treatment regimens. However, the conclusions need to be further validated in real-world prospective clinical trials.

## Materials and methods

### Data sources

The transcriptome expression data, mutation data and relevant clinicopathological parameters of patients with HCC in the TCGA-LIHC cohort were downloaded from the TCGA repository (https://portal.gdc.cancer.gov/). Strawberry Perl was used for transcriptomic and clinical data collation. Transcriptome data and relevant clinicopathological data of the independent validation cohort (GSE14520) were obtained from the Gene Expression Omnibus (GEO) database (https://www.ncbi.nlm.nih.gov/). Additionally, immune-related genes were obtained from the ImmPort database (https://www.immport.org/shared/home).

### Immune cluster analysis of HCC based on ssGSEA

Gene set enrichment analysis (GSEA) is a computational method that classifies sets of genes with common functions (Subramanian et al., 2005), whereas ssGSEA analyses the absolute enrichment of one gene set per sample within a given data set (Barbie et al., 2009). In the present study, ssGSEA was used to generate the enrichment fraction of 29 immune cells in each sample (Chen et al., 2022). The patients in the TCGA cohort were further classified into low- and high-immune clusters using cluster analysis. In this process R packages ‘GSVA’, ‘limma’, ‘GSEABase’, ‘sparcl’ for cluster analysis, ‘Rtsne’ package for principal component analysis (PCA) and ‘ggplot2’ for the visualisation of the results were used.

### Correlation analysis of immune clusters and TIME

ESTIMATE is an expression data-based tumour purity determination algorithm that predicts the level of infiltrating stromal cells and immune cells (Yoshihara et al., 2013). First, the R package ‘ESTIMATE’ was used to calculate the number of stromal and immune cells in the tumour tissue of each HCC case in the TCGA cohort. The total of the immune and stromal scores is the ESTIMATE scores were inversely related to tumour purity. Furthermore, the R package ‘reshape2’ was utilised to reconstruct the data, and the ‘pheatmap’ and ‘ggpubr’ packages were used to draw heat maps and violin maps, respectively, of stromal cells, immune cells and ESTIMATE scores in the high- and low-immune clusters.

CIBERSORT implements a machine-learning algorithm for the high-throughput characterization of different cell types (Newman et al., 2015). The fractions of the 22 tumor-infiltrating immune cells were determined by the R packages “CIBERSORT”, “preprocessCore”, “e1071” and “parallel” and further analyzed for differences in tumour-infiltrating immune cells (TIICs) in the two immune clusters.

Moreover, we further analysed the status of human leukocyte antigen (HLA) and immune checkpoint-related genes in the high- and low-immune typing groups, and the packages ‘ggplot2’ and ‘ggpubr’ were employed to visualize the results.

### Identification of differentially expressed immune-related genes in the two immune clusters

The ‘limma’ package identified differentially expressed genes (DEGs) between different immunophenotypes of HCC tumours (fold change (FC) > 1.5, false discovery rate (FDR) < 0.05). The ‘ggplot2’ and ‘pheatmap’ packages were used to generate volcano maps and expression heat maps of DEGs, respectively. The ‘venn’ package was used to map Venn diagrams to identify shared genes between DEGs and immune-related genes.

Additionally, gene ontology (GO) analysis of DEGs was performed using the R package ‘clusterProfiler’, ‘org.Hs.eg.db’ and ‘DOSE’. Furthermore, the ‘ggplot2’, ‘circlize’, ‘RColorBrewer’, ‘ggpubr’ and ‘ComplexHeatmap’ packages were employed to map enrichment outcomes and explore the enrichment of DEGs in cellular components, molecular function and biological processes.

### Construction of an IRGs in HCC

The R packages ‘glmnet’, ‘timeROC’, ‘survival’, ‘survminer’ and ‘caret’ were employed to obtain prognosis-related genes and construct IRGs. First, univariate Cox regression analysis screened DEGs associated with survival (*p* < 0.05) for further analysis. The LASSO algorithm was performed on the univariate prognostic genes to screen the optimal genes to construct the model. Risk scores for all cases were calculated using the following formula: $Risk\;scores=\overset{n}{\underset{i=1}{\sum }}Coefficient\left(i\right)*Expression\left(i\right)$ . Expression(i) and Coefficient(i) indicate the expression values and regression coefficient for each signature gene, respectively. Patients with HCC in the TCGA training cohort and GEO independent validation cohort were categorised into low- and high-risk groups based on the median risk scores.

### Evaluation of the IRGs in HCC

Survival analysis was performed using the ‘survivor’ and ‘survminer’ packages for the high- and low-risk groups. Moreover, risk curves, risk heat maps, survival curves and survival status plots were created for patients in the TCGA and GEO cohorts. Univariate and multivariate Cox regression analyses were used to determine the prognostic potential of the risk score of the signature. Time-dependent receiver operating characteristics (ROC) analyses were performed using the ‘survminer’, ‘survivor’ and ‘timeROC’ packages, which assessed the prognostic predictive value of the developed signatures. Additionally, Kaplan–Meier (KM) curves were used to analyse survival differences between the high- and low-risk subgroups based on different clinical characteristics (age, gender, tumour stage and tumour grade), thereby determining whether the developed IRGs applied to patients with HCC with different clinicopathological parameters. The ‘ComplexHeatmap’ was employed to draw the status heat map for high- and low-risk groups and clinicopathological parameters. The ‘ggpubr’ package was used to draw box plots of risk scores for different clinical subgroups to identify the correlation of the developed IRGs with different clinicopathological parameters.

### Correlation between signature and TMB

The downloaded mutation data were collated using the Strawberry Perl script to generate TMB data for each HCC sample. The package ‘Limma’ was used to analyse the TMB differences between the different risk groups, and the results were plotted using ‘ggpubr’. Additionally, the optimal cut-off values of TMB were obtained using the R software, and patients were further divided into low- and high-TMB groups. Furthermore, the ‘survivor’ and ‘survminer’ were employed to generate the K-M curves of patients in the high- and low-TMB groups combined with the high- and low-risk groups. Finally, the ‘maftools’ package was employed to plot the mutation waterfalls of the 20 genes with the most frequent mutations.

### GSVA

GSVA is a computational method used to detect pathway activity in a sample population (Hänzelmann et al., 2013). The GSVA analysis based on R software provides the enrichment of the Kyoto Encyclopedia of Genes and Genomes (KEGG) pathway in different risk groups and analyses the correlation between the KEGG pathway and signature gene mRNA expression. Accordingly, R packages ‘limma’, ‘reshape2’, ‘ggplot2’, ‘GSVA’, ‘GSEABase’ and ‘pheatmap’ were used.

### Nomogram construction

Tumour stage and risk scores were used to construct nomograms for 1-, three- and 5-year overall survival (OS) based on independent prognostic analysis results. Calibration curves for Hosmer–Lemeshow test were drawn (method = ‘boot’, B = 1,000) to evaluate if the predicted outcomes of the nomogram were in good agreement with the reality. This process utilised the ‘survival’, ‘regplot’ and ‘rms’ packages.

### Drug sensitivity analysis

To explore the potential clinical significance of the IRGs in drug therapy, the ‘pRRophetic’ package was employed to obtain the IC50 of different therapeutic agents in the high- and low-risk groups (Chen et al., 2021), and box plots were drawn using the ‘ggpubr’ for drugs with differences in IC50 (*p* < 0.001).

### Statistical analysis

R software was used to perform all statistical analyses. The significance of differences between K-M survival curves was determined using the log-rank test. Additionally, two-tailed *p*-values <0.05 were considered significant.

## Results

### Construction of HCC clustering based on ssGSEA

**FIGURE 1:**
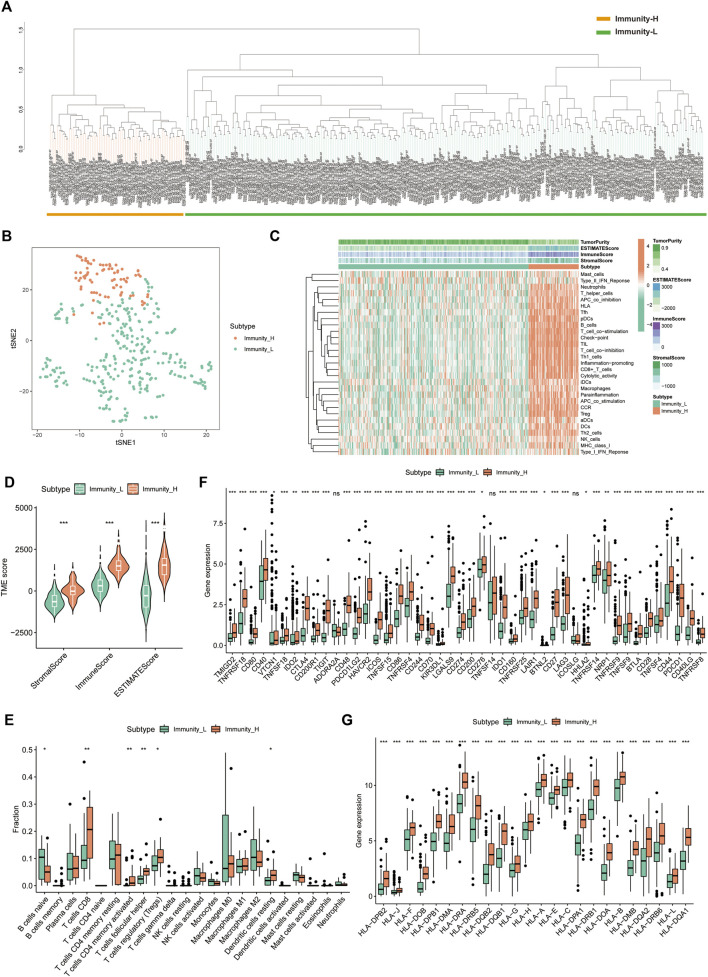
Single sample gene set enrichment analysis -based immune cluster analysis. **(A)** The hepatocellular carcinoma samples from The Cancer Genome Atlas cohort were divided into a high immune cell infiltration cluster (orange) and a low immune cell infiltration cluster (green). **(B)** The principal component analysis plot of the distribution status of the high- and low-immune clusters. **(C)** Enrichment levels of different types of immune cells in the high- and low-immune clusters. Tumour purity, ESTIMATE score, immune score and stromal score are displayed for each sample in combination with clustering information. **(D)** Violin plots of ESTIMATE score, immune score and stromal score in different immune clusters. **(E)** The box plot shows the difference in immune cell infiltration between the two clusters based on the CIBERSORT algorithm. **(F,G)** Box plots show the differences in the expression of immune checkpoint-related genes and HLA family genes in the two clusters, respectively. *p < 0.05, **p < 0.01 and ***p < 0.001.,

A total of 374 HCC tumour samples were obtained from the TCGA database for transcriptomic data. The level of infiltration of 29 immune cells in each HCC sample was obtained using ssGSEA. Patients were further divided into low- (*n* = 296) and high-immune clusters (*n* = 78) using a hierarchical clustering algorithm (Figures 1A,B). The heat map suggested that most immune cells infiltrate at higher levels in the high-immune cluster than in the low-immune cluster (Figure 1C). Furthermore, violin plots showed that all three scores were lower in the low-immune cluster than in the high-immune cluster (*p* < 0.001) (Figure 1D), which was consistent with the results of ssGSEA. This also reflected the low level of tumour purity in the high-immune cluster. Additionally, CIBERSORT analysis showed that the proportion of CD8^+^ T Cells, activated memory CD4^+^ T Cells, T follicular helper cells, resting dendritic cells and regulatory cells (Tregs) were higher in the high-immune cluster than in the low-immune cluster (*p* < 0.05) (Figure 1E). Box plots revealed that the expression of most immune checkpoint-related genes and HLA family-related genes were also significantly higher in the high-immune cluster than in the low-immune cluster (Figures 1F,G). Thus, the above results highlight the reliability of the ssGSEA-based immune clusters.

### Exploration of DEGs between the high- and low-immune clusters

**FIGURE 2:**
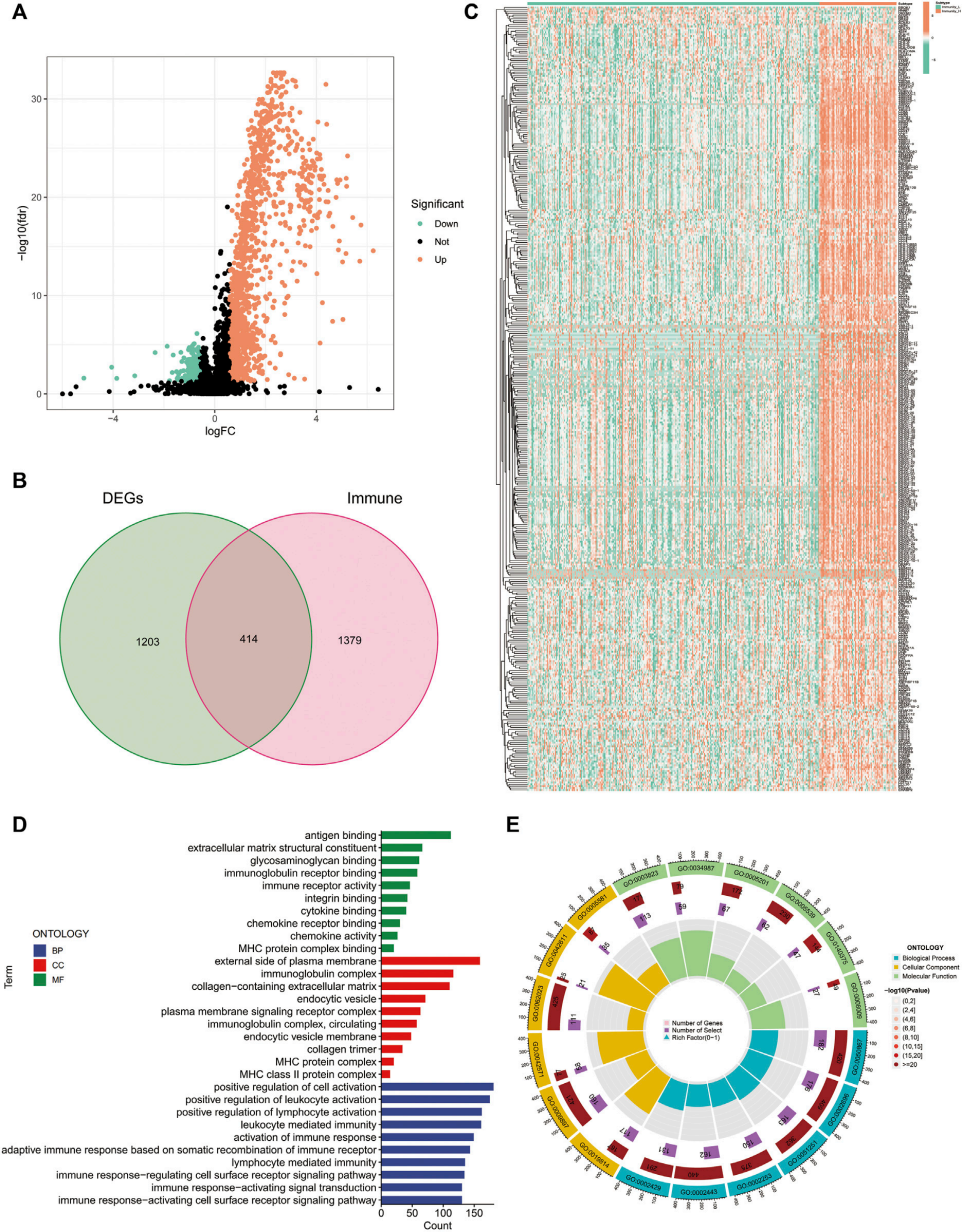
Differentially expressed genes between the high and low-immune clusters. **(A)** Volcano map of differentially expressed genes between the high- and low-immune clusters. **(B)** The Venn diagram shows the 414 intersecting genes that were obtained after intersecting the differentially expressed genes and immune-related genes. **(C)** Heat map of the expression of 414 intersecting genes in different immune clusters. **(D,E)** Enrichment of differentially expressed genes between the high- and low-immune clusters in terms of biological function *via* GO analysis.

Using differential analysis, we obtained 1,617 DEGs between the high- and low-immune clusters, of which 246 were down-regulated and the remaining 1,371 were up-regulated in the high-immune cluster (Figure 2A). After intersecting the DEGs with the 1793 immune-related genes derived from the ImmPort database, we obtained 414 immune-related DEGs (Figure 2B). The heat map displays the expression of the immune-related DEGs in the high- and low-immune clusters (Figure 2C).

Additionally, we also explored the biological functions of DEGs in the different immune clusters using GO analysis. In terms of biological processes, the DEGs were enriched in antigen binding, extracellular matrix structural constituent, glycosaminoglycan binding, immunoglobulin receptor binding, immune receptor activity and other processes. Regarding cellular components, the DEGs were enriched in the external side of the plasma membrane, immunoglobulin complex, MHC protein complex and MHC class II protein complex. Additionally, DEGs were also enriched in the positive regulation of leukocyte activation, positive regulation of lymphocyte activation, leukocyte-mediated immunity, activation of immune response and other molecular functions (Figures 2D, E).

### Construction of the IRGs in HCC

**FIGURE 3:**
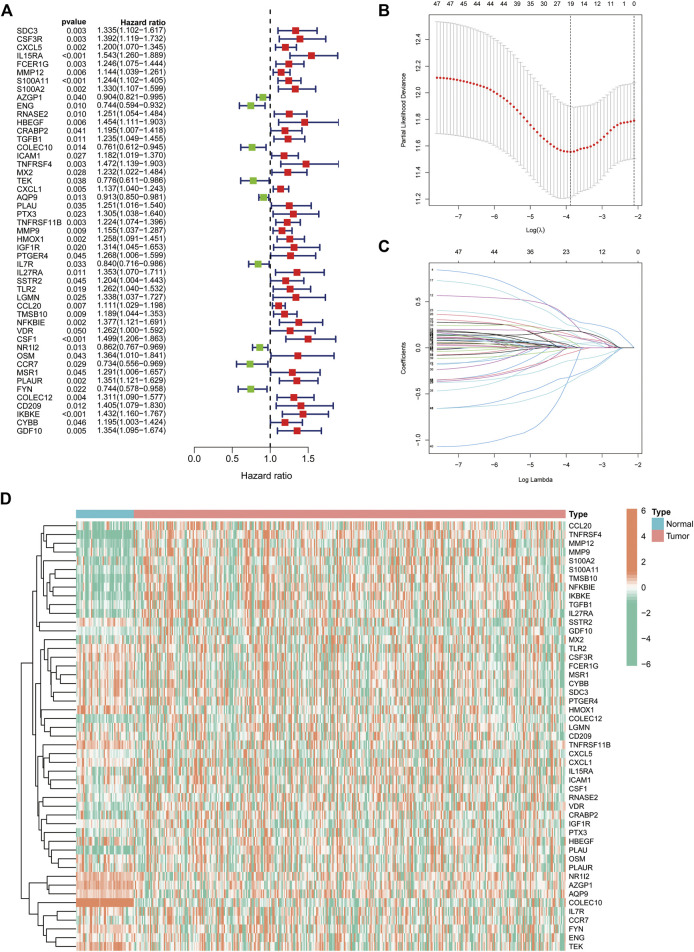
Identification of immune-related differentially expressed genes (DEGs) associated with prognosis for signature construction. **(A)** Forest plot of 49 immune-related DEGs significantly associated with overall survival in patients with hepatocellular carcinoma (HCC). **(B,C)** LASSO coefficient and partial likelihood deviance of the prognostic signature. **(D)** Heat map of 49 prognosis-related genes expressed in HCC tumour samples and normal samples.

We extracted 49 genes significantly associated with survival (*p* < 0.05) using Cox regression analysis of the 414 immune-related DEGs, of which nine were favourable prognosis genes (Figure 3A). The heat map displays the expression of 49 prognosis-related genes in HCC tumour samples and normal samples (Figure 3D). Furthermore, LASSO regression analysis on prognosis-related DEGs revealed 19 genes for signature construction (Figures 3B,C). We calculated risk scores for each patient with HCC based on the risk coefficients and expression of the 19 genes screened (Table 1). All patients were divided into high-risk and low-risk groups based on risk scores.

**TABLE 1:** Immune-associated signature genes.

Genes	Coef	HR	HR (95%CI)	*p*-value
IL15RA	0.318	1.543	1.260-1.889	<0.001
ENG	−0.314	0.744	0.594-0.932	0.010
RNASE2	0.061	1.251	1.054-1.484	0.010
HBEGF	0.298	1.454	1.111-1.903	0.006
TNFRSF4	0.241	1.472	1.139-1.903	0.003
PTX3	0.168	1.305	1.038-1.640	0.023
TNFRSF11B	0.120	1.224	1.074-1.396	0.003
HMOX1	0.141	1.258	1.091-1.451	0.002
IL7R	−0.137	0.840	0.716-0.986	0.033
TLR2	0.003	1.262	1.040-1.532	0.019
LGMN	0.074	1.338	1.037-1.727	0.025
CCL20	0.013	1.111	1.029-1.198	0.007
VDR	0.025	1.262	1.000-1.592	0.050
NR1I2	−0.006	0.862	0.767-0.969	0.013
OSM	−0.288	1.364	1.010-1.841	0.043
CCR7	−0.365	0.734	0.556-0.969	0.029
FYN	−0.055	0.744	0.578-0.958	0.022
IKBKE	0.027	1.432	1.160-1.767	<0.001
GDF10	0.185	1.354	1.095-1.674	0.005

HR, hazard ratio; CI, confidence interval.

### Validation of the IRGs in HCC

**FIGURE 4:**
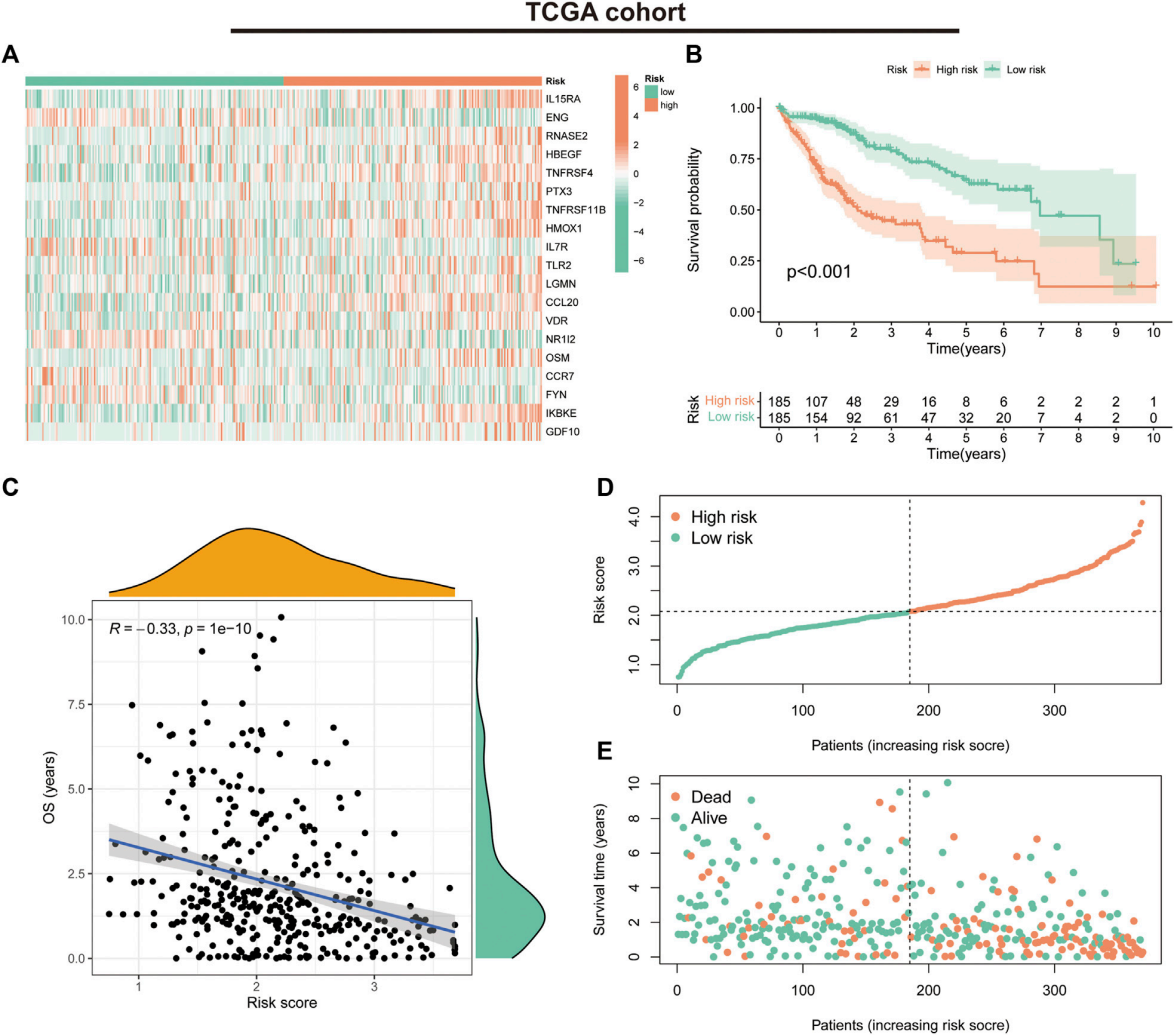
Prognostic values of the immune-related gene signature in The Cancer Genome Atlas (TCGA) cohort. **(A)** Heat map showing expression levels of the 19 immune-related genes in the TCGA cohort. **(B)** Kaplan–Meier curve for overall survival (OS) in the TCGA cohort. **(C)** Scatter plot of correlation between risk score and OS in the TCGA cohort. **(D)** Risk score distribution in the TCGA cohort. **(E)** Survival time and status in the TCGA cohort.

We first evaluated the prognostic predictive value of the signature in the TCGA training cohort. The heat map shows the expression status of the 19 genes in the risk groups (Figure 4A). K-M survival curves suggested that patients in the low-risk group had significantly better survival than those in the high-risk group (*p* < 0.001) (Figure 4B). Correlation analysis suggested a negative correlation between risk scores and OS (correlation coefficient = -0.33, *p* < 0.001) (Figure 4C). Further evaluation of the survival status and risk score distribution of patients revealed that the low-risk group had a better prognosis than the high-risk group (Figures 4D, E).

**FIGURE 5:**
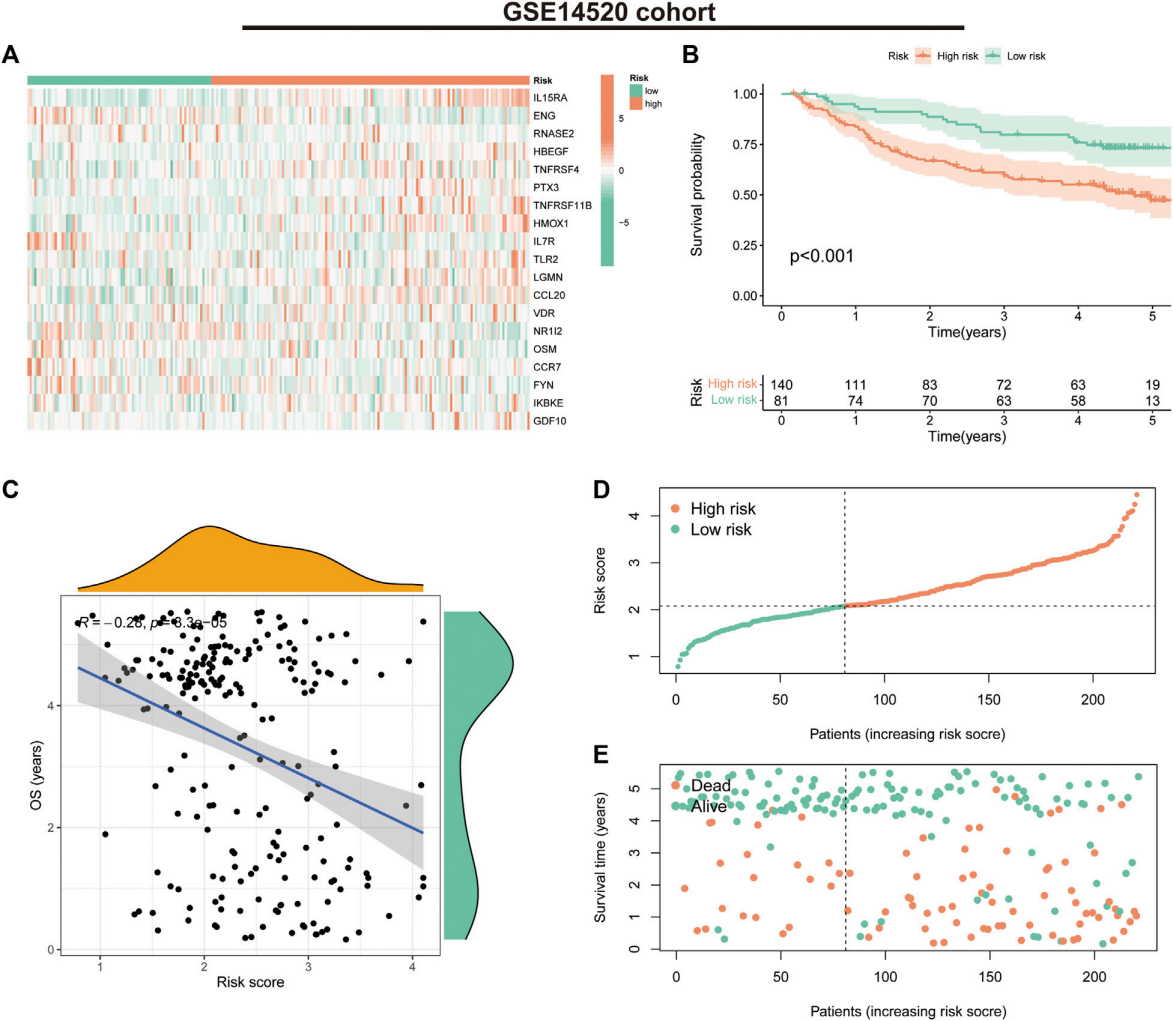
Prognostic values of the immune-related gene signature in the GSE14520 validation cohort. **(A)** Heat map showing expression levels of the 19 immune-related genes in the validation cohort. **(B)** Kaplan–Meier curve for overall survival (OS) in the validation cohort. **(C)** Scatter plot of correlation between risk score and OS in the validation cohort. **(D)** Risk score distribution in the validation cohort. **(E)** Survival time and status in the validation cohort.

In the GSE14520 validation cohort, we also observed a trend in the expression of the 19 risk model genes in the risk groups (Figure 5A). K-M survival curves suggested that patients in the low-risk group had significantly better survival than those in the high-risk group (*p* < 0.001) (Figure 5B). The correlation analysis suggested a negative correlation between risk scores and OS (correlation coefficient = -0.28, *p* < 0.001) (Figure 5C). Furthermore, the survival status and risk score distribution of patients in the different risk groups in the validation cohort also suggested that the patients with HCC in the low-risk group had a better prognosis than those in the high-risk group (Figures 5D, E). These findings highlight the validity and stability of the IRGs constructed based on the LASSO algorithm.

**FIGURE 6:**
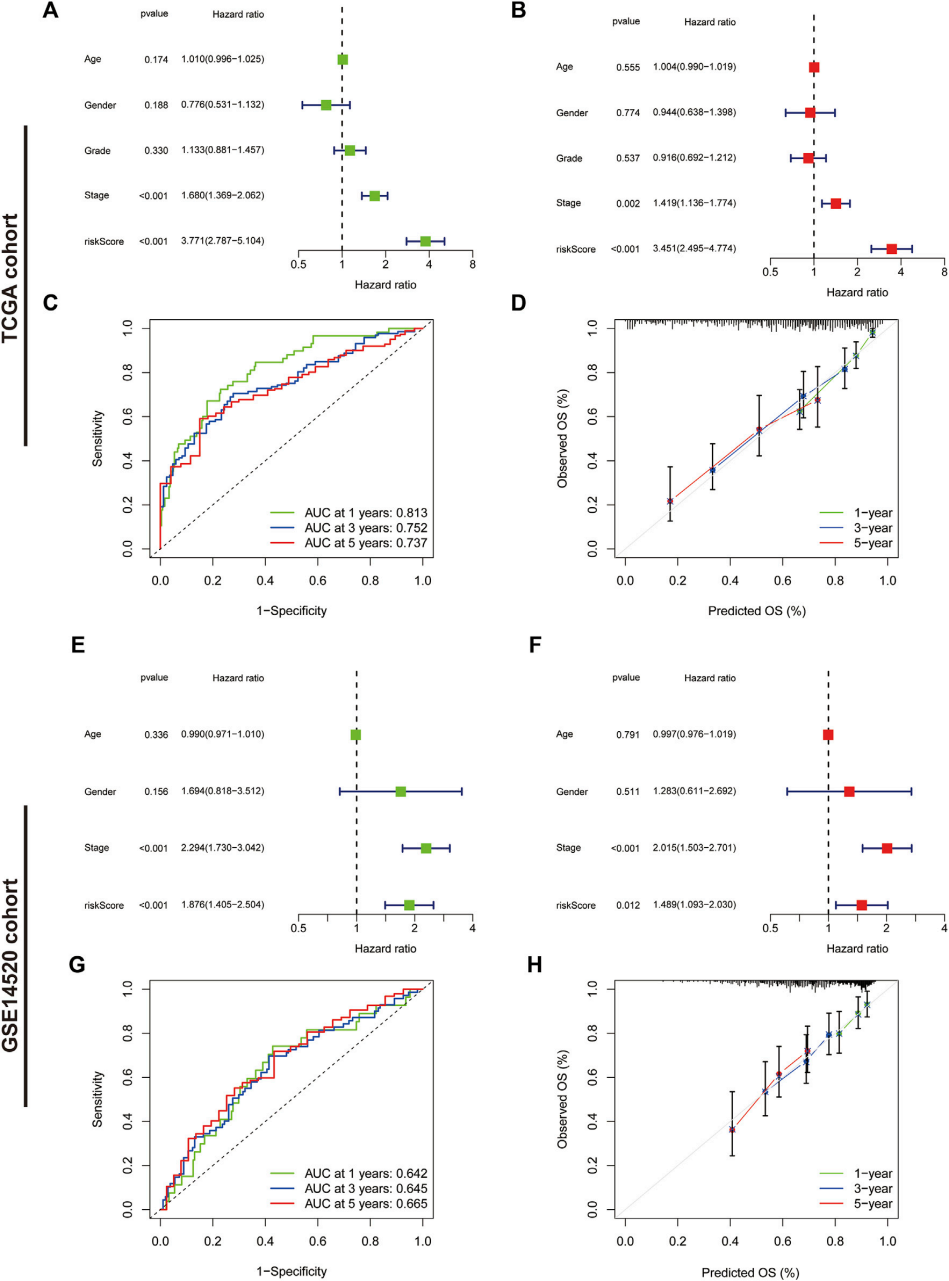
Assessment of the immune-related gene signature. **(A)** Forest plot for univariate Cox and **(B)** multivariate Cox regression analysis in The Cancer Genome Atlas (TCGA) cohort. **(C)** Receiver operating characteristic (ROC) curves of 1-, three- and 5-year survival for the predictive signature in the TCGA cohort. **(D)** The calibration curves for 1-, three- and 5-year OS in the TCGA cohort. **(E)** Forest plot for univariate Cox and **(F)** multivariate Cox regression analysis in the validation cohort. **(G)** ROC curves of 1-, three- and 5-year survival for the predictive signature in the validation cohort. **(H)** The calibration curves for 1-, three- and 5-year overall survival in the validation cohort.

We further assessed the prognostic predictive efficacy of the IRGs using COX regression analyses and ROC curves, which revealed that the risk score was an independent prognostic indicator, with a hazard ratio (HR) of 3.771 and 3.451 in the univariate and multifactorial Cox analyses, respectively (*p* < 0.001) (Figures 6A,B). Additionally, the tumour stage was considered an independent prognostic factor with an HR of 1.680 (*p* < 0.001) and 1.419 (*p* = 0.002). The area under the ROC curve for risk score at 1-, three- and 5-year was 0.813, 0.752 and 0.737, respectively (Figure 6C), and the calibration curves suggested a good agreement between the survival prediction results of risk score and the actual outcome (Figure 6D). In the GSE14520 cohort, risk score and tumour stage were also independent prognostic predictors (Figures 6E, F). The area under the ROC curve at 1-, three- and 5-year was 0.642, 0.645 and 0.665, respectively (Figure 6G). Moreover, the calibration curve suggested good prognostic predictive efficacy of risk score (Figure 6H).

### Correlation of the IRGs with clinicopathological parameters in HCC

**FIGURE 7:**
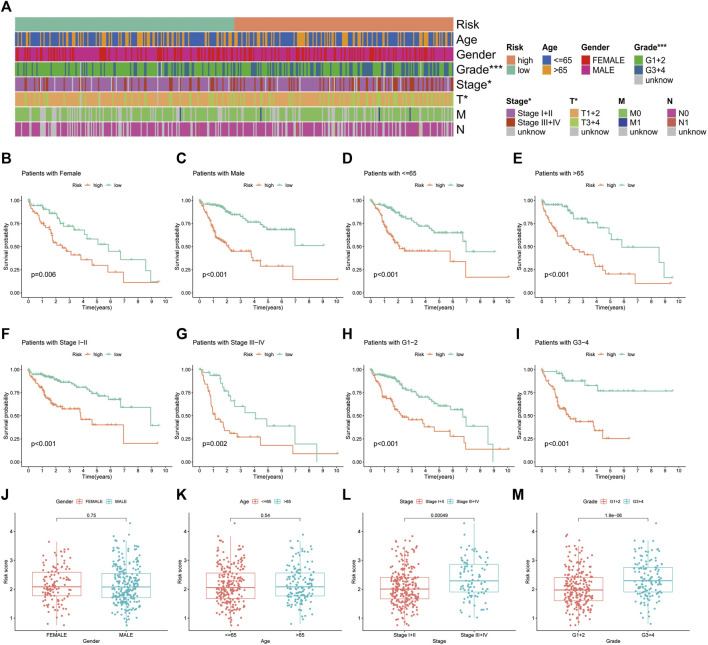
Correlation analysis of the immune-related gene signature with clinicopathological parameters in The Cancer Genome Atlas cohort. **(A)** Heat map of the distribution of clinicopathological parameters in the high- and low-risk groups. **(B,C)** Kaplan–Meier survival curves of low- and high-risk groups sorted by gender, **(D,E)** age, **(F,G)** TNM stage and **(H,I)** tumour grade. Different levels of risk scores in patients with hepatocellular carcinoma were stratified by **(J)** gender, **(K)** age, **(L)** TNM stage and **(M)** tumour grade.

The heat map illustrates the status of different clinicopathological parameters in the risk groups (Figure 7A). Stratified K-M curves suggested that patients with HCC having different gender, age, tumour stage and grade had worse survival in the high-risk group than in the low-risk group (Figures 7B–I), demonstrating the stability and wide applicability of the IRGs. Moreover, analysis of risk scores in different clinicopathological parameters revealed lower risk scores in tumour Stage I + II and grade G1+2 than in Stage III + IV and grade G3+4, without significant differences in age and gender (Figure 7J–M).

### Nomogram construction and validation in HCC

**FIGURE 8:**
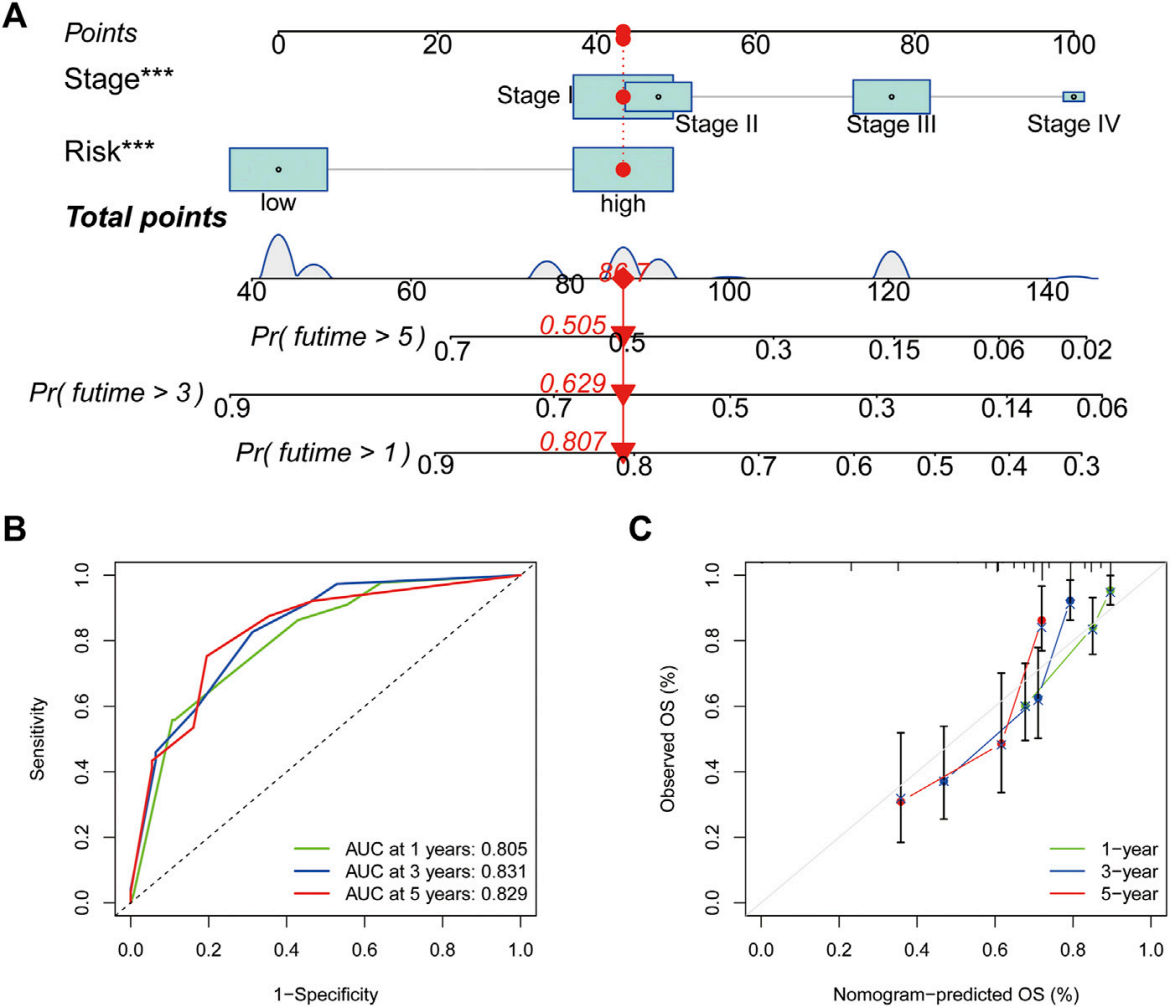
Nomogram construction and assessment. **(A)** Nomogram for predicting the 1-, three- and 5- years survival of patients with hepatocellular carcinoma. **(B)** Receiver operating characteristic curves of 1-, three- and 5-year survival for the predictive Nomogram. **(C)** The calibration curves for 1-, three- and 5-year overall survival. *p < 0.05, **p < 0.01 and ***p < 0.001.

Based on the outcomes of Cox regression, risk scores and tumour stage were used to construct a nomogram to predict the prognosis of patients with HCC (Figure 8A). The corresponding scores of tumour stage and risk score in the nomogram were calculated and the sum of the two was used as a predictive tool for prognosis. The area under the ROC curve for the 1-, three- and 5-year OS was 0.805, 0.831 and 0.829, respectively (Figure 8B). The calibration curves indicated the good predictive efficacy of the nomogram (Figure 8C).

### Correlation of the IRGs with TMB in HCC

**FIGURE 9:**
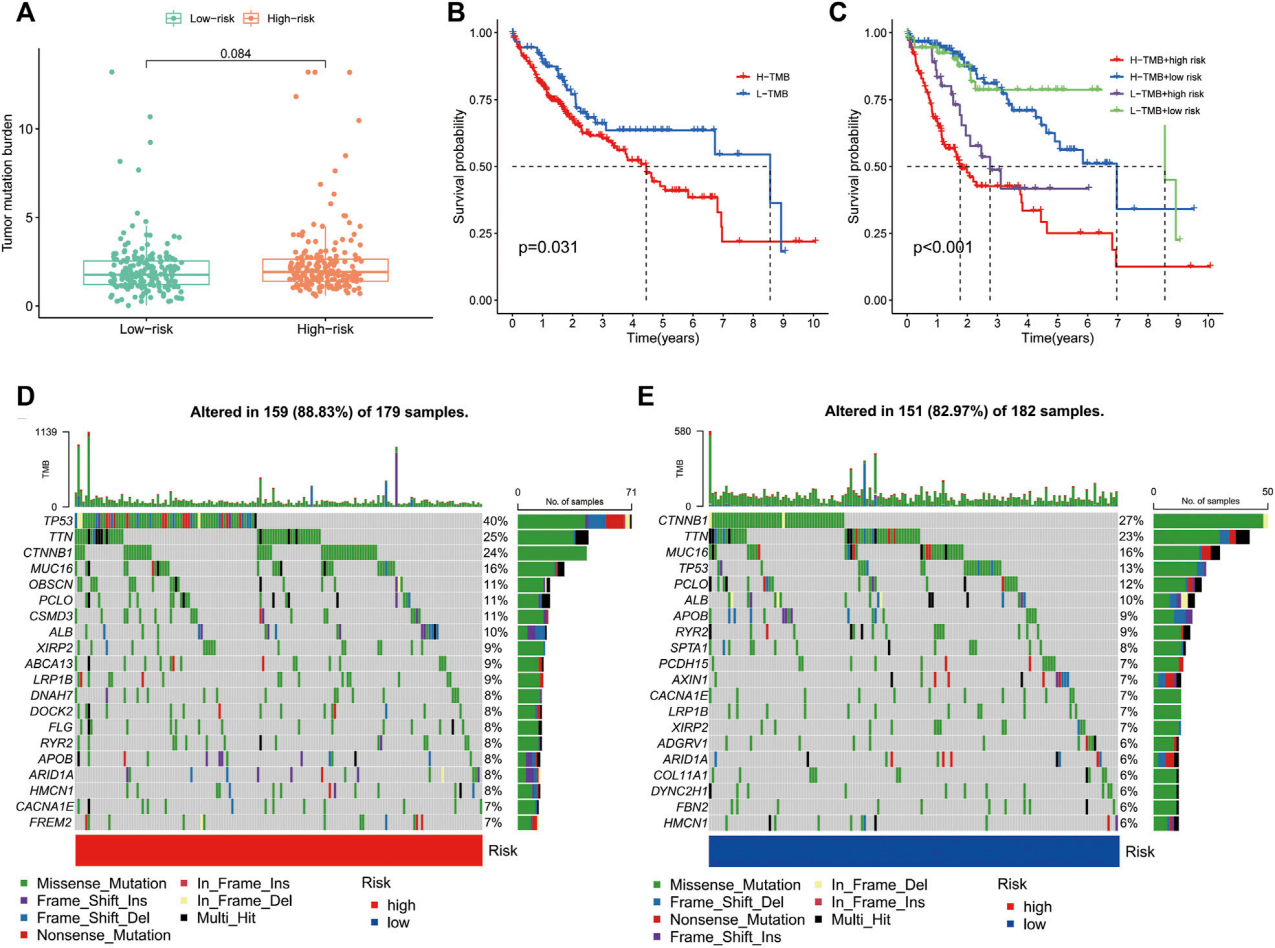
Correlation of the immune-related gene signature with tumour mutation burden (TMB) in hepatocellular carcinoma. **(A)** Violin plot of TMB in the high- and low-risk groups. **(B)** Kaplan–Meier curve of high-TMB and low-TMB. **(C)** Kaplan–Meier curve of the patients in the high- and low-TMB groups combined with the high- and low-risk groups. **(D)** Mutant gene waterfall plot in the high- and **(E)** low-risk groups.

TMB is the number of somatic non-synonymous mutations in a given genomic region and can indirectly reflect the capacity and extent of neoantigen generated by tumours and predict the effectiveness of immunotherapy for some tumours (Chan et al., 2019). Box plots revealed no significant difference in TMB levels between the high- and low-risk groups (Figure 9A). KM curves showed that higher TMB in HCC was associated with a poorer OS (Figure 9B). Notably, survival analysis showed significant differences between the four groups of high-risk/high-TMB, low-risk/low-TMB, high-risk/low-TMB and low-risk/high-TMB (*p* < 0.001), with the worst OS observed in the high-TMB/high-risk group and the best OS observed in the low-TMB/low-risk group (Figure 9C). Additionally, the mutation frequency in the high-risk group was 88.83% compared to 82.97% in the low-risk group (Figures 9D, E). In the high-risk group, the genes with the highest mutation frequencies were *TP53* (40%), *TTN* (25%) and *CTNNB1* (24%), whereas *CTNNB1* (27%), *TTN* (23%) and *MUC16* (16%) were observed in the low-risk group.

### GSVA of the IRGs in HCC

**FIGURE 10:**
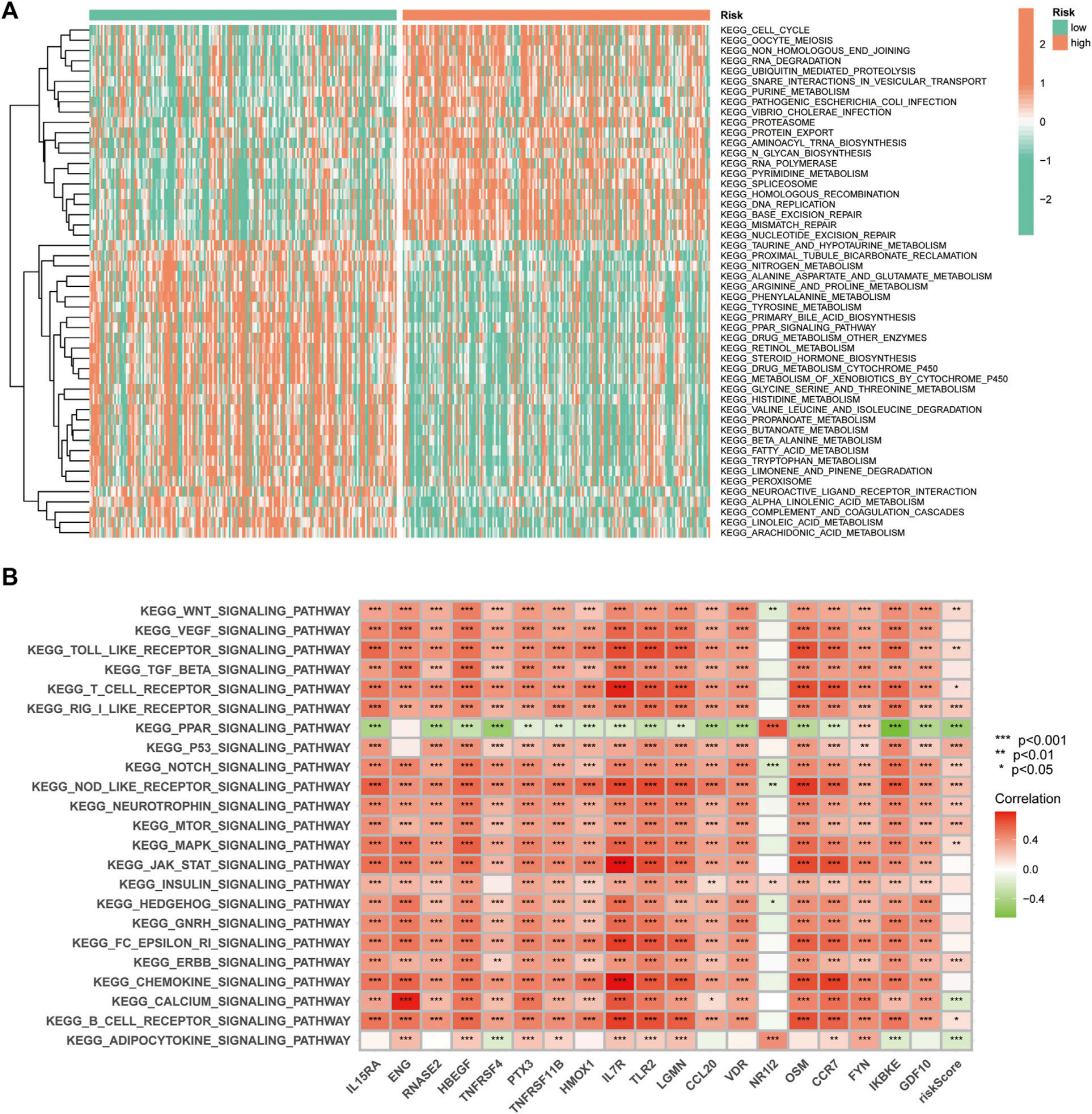
The Gene Set Variation Analysis. **(A)** Heat map highlighting the differences in functional pathways in the high- and low-risk groups. **(B)** The correlation between the KEGG pathway and signature gene mRNA expression.

GSVA was used to explore the differences in biological behaviour between the two risk groups. The high-risk group was enriched in pathways related to ubiquitin-mediated protein hydrolysis, cell cycle, protein export, RNA polymerase, DNA replication, homologous recombination, mismatch repair and nucleotide excision repair, which are associated with tumour biological behaviour. Moreover, functions such as nitrogen metabolism, fatty acid metabolism and multiple amino acid metabolisms were enriched in the low-risk group (Figure 10A). Additionally, we analysed the correlation between the 19 genes in the signature and different signalling pathways. Furthermore, a broad correlation between the expression of the IRGs and tumour-related signalling pathways was observed (Figure 10B).

### Drug sensitivity in the risk groups

**FIGURE 11:**
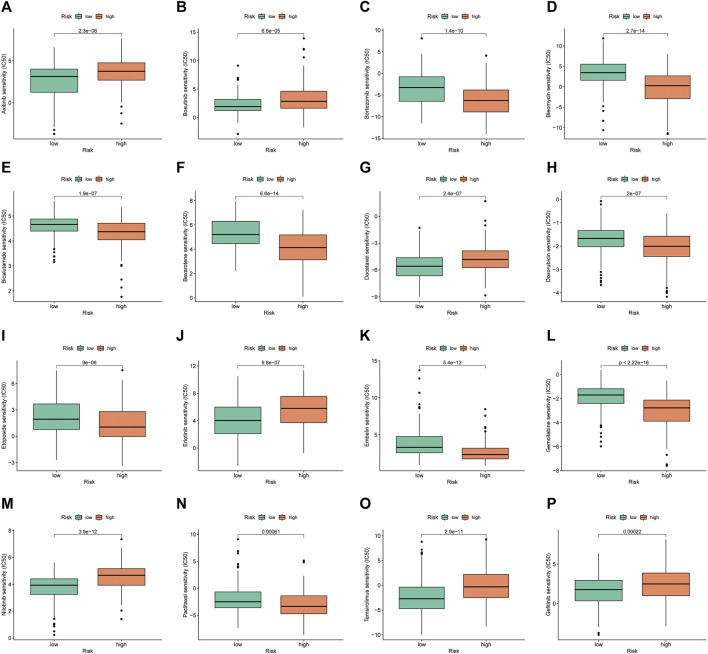
Investigation of drug sensitivity in risk groups. **(A–P)** Comparison of IC50 values for different agents in the high- and low-risk groups.

Drug sensitivity analysis showed differences in IC50 values between the different chemical and targeted agents in the different risk groups (*p* < 0.001) (Figure 11A-P). Notably, the IC50 values of the targeted therapeutics axitinib, bosutinib, erlotinib, nilotinib and gefitinib were lower in the low-risk group than in the high-risk group, suggesting that patients with HCC in the low-risk group may be more sensitive to small molecule targeted therapeutics. However, contrary results were observed for most chemotherapeutic agents including doxorubicin, bleomycin, etoposide, gemcitabine and paclitaxel, suggesting that high-risk patients may benefit more from chemotherapy.

## Discussion

HCC is one of the most common malignancies and is characterized by high aggressiveness, a tendency to metastasis and frequent recurrence (Llovet et al., 2021; Sung et al., 2021). Although recent improvements and optimizations of comprehensive treatment modalities, including surgery, interventional therapy, radiotherapy, chemotherapy, targeted therapy and immunotherapy, have been reported, the high degree of heterogeneity and poor prognosis of HCC remains an insurmountable problem.

Increasingly studies report that TIME has a significant impact on the occurrence, progression, treatment response and long-term prognosis of patients with HCC (Kurebayashi et al., 2018; Llovet et al., 2022). Additionally, with the rapid development of immunotherapy in the treatment of solid tumours, its use in HCC has garnered increasing attention. However, due to the low overall efficiency and response rate of immunotherapy, the superior population or prediction system for immunotherapy requires further exploration. Although the predictive value of PD-L1 expression, a classical efficacy prediction marker for ICIs, has been extensively evaluated in many tumour types (Reck et al., 2019; Paz-Ares et al., 2020), its predictive value for patients with HCC treated with ICIs remains unexplored (Macek Jilkova et al., 2019). TMB has also been reported as a potential predictor of the efficacy of ICIs in non-small cell lung cancer, melanoma and other malignancies (Chan et al., 2019; Samstein et al., 2019). However, it is unclear whether TMB in HCC can influence the response to ICIs owing to limited data (Shrestha et al., 2018; Rizzo and Ricci, 2022). Microsatellite instability (MSI) degree is another potential predictor of ICIs treatment (Chang et al., 2018). Theoretically, MSI-high degree (MSI-H) increases neoantigens, leads to effector lymphocyte activation and increases tumour sensitivity to ICIs (Svrcek et al., 2019). Non-etheless, data on the value of this predictor in HCC are scarce, with MSI-H being reported in less than 3% of patients (Goumard et al., 2017; Rizzo and Ricci, 2022). As TIME is constructed by tumour cells in conjunction with immune cells in the tumour microenvironment, it may be theoretically difficult for individual indicators to accurately predict patient prognosis and immunotherapy efficacy. Therefore, understanding the characteristics of TIME and immune cell infiltration in HCC is essential for the development of novel and accurate prognostic and therapeutic efficacy predictive biomarkers.

In this study, an unsupervised hierarchical clustering method based on ssGSEA was employed to analyse the immune clusters of patients with HCC and classify them into high- and low-immune clusters, thereby identifying patients with HCC patients having different TIME characteristics and inferring their response to immunotherapy. The ESTIMATE and CIBERSORT algorithms further validated these findings, suggesting that stromal cells, immune cells and ESTIMATE scores were significantly higher in the high-immune cluster than in the low-immune cluster. Studies have shown that tumours characterised by high infiltration of effector immune cells such as CD8^+^ T Cells and activation of immune checkpoints are considered to be ‘hot immune tumours’, which benefit highly from ICI treatments (Galon and Bruni, 2019; Liu and Sun, 2021). Notably, most of the immune checkpoint-related genes, including *PD-L1, PD-1, CTLA-4* and *LAG3*, are more highly expressed in the high-immune clusters than in the low-immune clusters, suggesting that patients with HCC in the high-immune cluster fit the basic profile of an ‘immune hot tumour’ and are potential beneficiaries of treatment with ICIs. Additionally, polymorphisms in the HLA genes are speculated to be involved in biological behaviours such as the immune escape of tumours (Sabbatino et al., 2020). HLA class I has been reported to be highly expressed in cancer cells, which may contribute to the antitumour effects of cytotoxic T lymphocyte-based cancer immunotherapy (Akazawa et al., 2019). In this study, most of the HLA genes were also highly expressed in the high-immune cluster, supporting the conclusion that the high-immune cluster is more likely to benefit from treatment with ICIs than those in the low-immune cluster.

To further explore the predictive value of immune-related genes in the prognosis of HCC, we extracted 19 of these DEGs for the construction of IRGs based on the LASSO algorithm and classified all patients into high- and low-risk groups. We further validated this signature in the TCGA and GSE14520 cohorts *via* K-M survival curves, univariate and multivariate Cox regression analysis and ROC curves. The results indicated that the signature had a reliable and good prognostic predictive power and could be applied to patients with HCC having different clinicopathological parameters.

Currently, advanced HCC is treated systemically with chemotherapy, targeted therapies and immunotherapy; however, the survival benefit remains poor. Precise individualized treatment and a combination of different systemic therapies are the future trends in the management of advanced HCC. The IRGs constructed in this study provide the basis for the selection of some chemotherapeutic agents and targeted drugs. In a recent study, ICI avelumab in combination with the TKI axitinib showed antitumour activity with controlled toxicity in patients with advanced HCC (Kudo et al., 2021). Our results suggested that the low-risk group was more sensitive to axitinib than the high-risk group. Moreover, bevacizumab in combination with erlotinib was found to be effective in patients with sorafenib-resistant HCC (He et al., 2019). Similarly, in this study, the low-risk group benefited more from erlotinib-targeted therapy. Notably, consistent results were observed with small-molecule targeted therapeutics such as bosutinib, nilotinib and gefitinib. However, most chemotherapeutic agents, including doxorubicin, bleomycin, etoposide, gemcitabine and paclitaxel, had lower IC50 values in the high-risk group, suggesting that high-risk patients could be more sensitive to chemotherapeutic agents.

Although the IRGs was validated by different methods, there remain some limitations. First, in retrospective studies, there may be some bias in the included cases. Second, we only used GSE14520 cohort for external validation, whereas we still need data from our own clinical cohort of patients with HCC for prospective analysis to test the applicability of the predictive signature.

## Conclusion

The immune cluster analysis based on the ssGSEA algorithm could effectively predict the immune microenvironment characteristics of patients with HCC and distinguish between ‘hot immune tumours’ and ‘cold immune tumours’, which could provide a basis for the selection of treatment for ICIs. Additionally, the IRGs constructed based on the LASSO algorithm was a good predictor of prognosis for patients with HCC, which could guide the selection of personalised treatment regimens. In the future, we will conduct prospective studies to further validate our results in clinical patients with HCC.

## Data Availability

The original contributions presented in the study are included in the article/Supplementary Material further inquiries can be directed to the corresponding author.
