# Supplementary material for: Comprehensive analysis of immune-related gene signature based on ssGSEA algorithms in the prognosis and immune landscape of hepatocellular carcinoma
Source: Front Genet. 2022 Dec 9;13:1064432. doi: 10.3389/fgene.2022.1064432 (PMC9780543; doi:10.3389/fgene.2022.1064432)
Supplement: Supplementary file 1 [file DataSheet1.ZIP › Supplementary Material/List of Supplementary Materials.docx]

# Supplementary Materials

1. Appendix 1. Clinical parameters of HCC patients in the TCGA cohort
2. Appendix 2. List of immune-related genes
3. Appendix 3. Clinical parameters of HCC patients in GEO cohort
4. Appendix 4. Tumor mutation burden data
5. Appendix 5. Results of ssGSEA analysis
6. Appendix 6. Results of immune cluster analysis
7. Appendix 7. Results of ESTIMATE analysis
8. Appendix 8. Results of CIBERSORT analysis
9. Appendix 9. Differentially expressed gene data between immune clusters
10. Appendix 10. Immune-related differentially expressed genes
11. Appendix 11. Data on the merging of expression and survival information of intersecting genes in the TCGA cohort
12. Appendix 12. Data on the merging of expression and survival information of intersecting genes in the GEO cohort
13. Appendix 13. Univariate Cox regression analysis data
14. Appendix 14. Regression coefficient data for model genes
15. Appendix 15. Risk scores for the TCGA cohort
16. Appendix 16. Risk scores for the GEO cohort
17. Appendix 17.Data from univariate and multivariate Cox regression analysis of the TCGA cohort
18. Appendix 18. Data from univariate and multivariate Cox regression analysis of the GEO cohort
19. Appendix 19. Risk data of nomograms
20. Appendix 20. Immune-related genes for ssGSEA
